# Vertical Intrauterine Bovine and Ovine Papillomavirus Coinfection in Pregnant Cows

**DOI:** 10.3390/pathogens13060453

**Published:** 2024-05-26

**Authors:** Francesca De Falco, Anna Cutarelli, Leonardo Leonardi, Ioan Marcus, Sante Roperto

**Affiliations:** 1 Dipartimento di Medicina Veterinaria e delle Produzioni Animali, Università degli Studi di Napoli Federico II, 80137 Naples, Italy; francesca.defalco@unina.it; 2 Area Science Park, Campus di Baronissi, Università degli Studi di Salerno, 84081 Baronissi, Italy; 3 Istituto Zooprofilattico Sperimentale del Mezzogiorno, 80055 Portici, Italy; annacutarelli@hotmail.it; 4 Dipartimento di Medicina Veterinaria, Università degli Studi di Perugia, 06126 Perugia, Italy; leonardi@unipg.it; 5 Faculty of Veterinary Medicine, University of Agricultural Sciences and Veterinary Medicine Cluj-Napoca, 400000 Cluj-Napoca, Romania; ioan.marcus@usamvcluj.ro

**Keywords:** bovine papillomavirus, bovine fetuses, ddPCR, ovine papillomavirus, transplacental coinfection

## Abstract

There is very little information available about transplacental infections by the papillomavirus in ruminants. However, recent evidence has emerged of the first report of vertical infections of bovine papillomavirus (BPV) in fetuses from naturally infected, pregnant cows. This study reports the coinfection of BPV and ovine papillomavirus (OaPV) in bovine fetuses from infected pregnant cows suffering from bladder tumors caused by simultaneous, persistent viral infections. Some molecular mechanisms involving the binary complex composed of Eras and platelet-derived growth factor β receptor (PDGFβR), by which BPVs and OaPVs contribute to reproductive disorders, have been investigated. A droplet digital polymerase chain reaction (ddPCR) was used to detect and quantify the nucleic acids of the BPVs of the *Deltapapillomavirus* genus (BPV1, BPV2, BPV13, and BPV14) and OaPVs belonging to the *Deltapapillomavirus* (OaPV1, OaPV2, and OaPV4) and *Dyokappapapillomavirus* (OaPV3) genera in the placenta and fetal organs (heart, lung, liver, and kidneys) of four bovine fetuses from four pregnant cows with neoplasia of the urinary bladder. A papillomaviral evaluation was also performed on the bladder tumors and peripheral blood of these pregnant cows. In all fetal and maternal samples, the genotype distribution of BPVs and OaPVs were evaluated using both their DNA and RNA. A BPV and OaPV coinfection was seen in bladder tumors, whereas only BPV infection was found in peripheral blood. The genotype distribution of both the BPVs and OaPVs detected in placentas and fetal organs indicated a stronger concordance with the viral genotypes detected in bladder tumors rather than in peripheral blood. This suggests that the viruses found in placentas and fetuses may have originated from infected bladders. Our study highlights the likelihood of vertical infections with BPVs and OaPVs and emphasizes the importance of gaining further insights into the mechanisms and consequences of this exposure. This study warrants further research as adverse pregnancy outcomes are a major source of economic losses in cattle breeding.

## 1. Introduction

Papillomaviruses are small, nonenveloped, double-stranded DNA viruses that infect the mucosal and cutaneous epithelia of vertebrates, resulting in benign and malignant lesions of the skin and mucosa [1].

Beyond cancer, the papillomavirus infection has increasingly gained attention because of its potentially harmful impact on reproductive performances. It has been shown that human papillomavirus (HPV), prevalent among pregnant women worldwide, is associated with decreased sperm motility and an increase in abortion rates [2,3,4,5,6]. As with many viral diseases, the impact of papillomaviral infections on pregnancy outcomes and reproductive performance in cows is still actually unknown.

Bovine papillomaviruses (BPVs) belonging to the *Deltapapillomavirus* genus, specifically types 1, 2, 13, and 14 (BPV1, BPV2, BPV13, and BPV14), are known to be highly pathogenic viruses [7]. They play a crucial role in carcinogenetic mechanisms, particularly in cattle that have grazed on lands rich in bracken fern (*Pteridium* spp.) [8,9,10]. Furthermore, observational studies suggest that infection by these viruses may affect other aspects of animal health, such as fertility [11,12]. BPVs have been shown to be responsible for fetal and placental infections, resulting in adverse reproductive outcomes in cattle [11]. Their DNA was found in the amniotic liquid and membranes, as well as placenta of pregnant cows [12,13,14,15]. Experimental studies suggest that these viruses can be vertically transmitted through the bloodstream [16]. It has been shown that Delta BPVs are transcriptionally active in the bovine placenta as both E5 oncoprotein and L1 protein have been detected in the placental trophoblast cells of pregnant cows, showing that trophoblast cells are an important target of BPV infection, ref. [15], similar to HPV that targets human trophoblast cells too [2,4]. Furthermore, Delta BPV DNA has also been detected in the placenta of mares [17]. Recently, evidence has been provided of the first prenatal viral vertical transmission in pregnant cows suffering from a persistent BPV infection associated with bladder tumors [11].

Ovine papillomaviruses (OaPVs—*Ovis aries Papillomaviruses*) are oncogenic viruses that comprise four genotypes. OaPV1, OaPV2, and OaPV4 are fibropapillomaviruses belonging to the *Deltapapillomavirus* genus, whereas OaPV3 is a *Dyokappapapillomavirus* [18]. All OaPV genotypes have a tropism limited to dermal fibroblast and keratinocyte cells only [19,20]. Thus, OaPVs have been linked to the development of cutaneous papillomas/fibropapillomas and squamous cell carcinomas in sheep [19,21,22,23]. Recently, OaPV DNA and RNA have been detected in the peripheral blood of cows, indicating that these viruses can be accountable for novel cross-species transmission [24]. Furthermore, all four OaPV genotypes have been found in a transcriptionally active form in BPV-negative bladder tumors of cattle, suggesting that OaPVs could be responsible for persistent cross-species urinary bladder infections resulting in bladder carcinogenesis [25]. There is still scant and very limited knowledge of the OaPV-related pathology. OaPVs have never been found in the organs of the male and/or female reproductive system. Therefore, whether OaPV infection may represent a risk and/or have a harmful potential effect on pregnancy and/or fertility is completely unknown.

It has been shown that a binary complex composed of the PDGFβR and ERAS is expressed in the tissues of adult cattle [26]. PDGFβR is a ligand of the PDGF signaling network in modulating cell proliferation, survival, and migration both during development and in the adult animal. Therefore, PDGFβR plays a crucial role in vascular and hematopoietic development, skeletal patterning, and organogenesis [27]. In particular, activated (phosphorylated) PDGFβR is involved in placental angiogenesis [28]. The PDGFβR expression is dynamic, being low in vivo during development but increasing dramatically during inflammation. Eras is a novel gene of the Ras family. Unlike humans, ERAS is constitutively expressed in adult tissues of domestic animals [26,29]. It has been suggested that ERAS has a role in the homeostasis of cells as it is a key player in basal autophagy [29].

The PDGFβR/ERAS complex appeared to be overexpressed in papillomavirus-associated tumors of the bovine urinary bladder [30]. Furthermore, it has been suggested that the major pathway by which bovine papillomaviral proteins display their activity is based on the activation of PDGFβR [31]. Therefore, we aimed to investigate these factors, including their downstream effector, the protein kinase, and AKT, in the placenta and fetal organs during the papillomavirus transplacental infection.

This study aimed to (I) provide the first evidence for a vertical intrauterine BPV and OaPV coinfection detected in fetuses of pregnant cows affected by BPV- and OaPV-associated bladder tumors, and (II) investigate some molecular mechanisms by which BPVs and OaPVs contribute to the causality of viral disease, including adverse pregnancy outcomes.

## 2. Materials and Methods

### 2.1. Ethics Statement

We did not perform any animal experiments during this study. All samples were collected postmortem from slaughterhouses, and thus this research did not require ethical approval.

### 2.2. Animal Samples

Four pregnant cows were slaughtered at public slaughterhouses after they suffered from chronic enzootic hematuria, a severe clinical syndrome caused mostly by papillomavirus-associated bladder tumors. Although reproductive history of pregnant cows was not available, morphological aspects of fetuses suggest that they were at midterm. Upon receiving permission from the medical authorities, we collected blood, bladder, and placenta samples from these animals. Furthermore, we were able to collect liver, kidney, heart, and lung samples from the midterm fetuses of these affected cows. Each sample was immediately divided into several parts that were frozen in liquid nitrogen for subsequent molecular biological analysis or fixed in 10% neutral buffered formalin (bladder samples only) and processed for paraffin embedding for microscopic investigation. Histological assessment of bladder tumors was performed on 5 µm-thick hematoxylin–eosin (HE)-stained sections, following the suggested morphological criteria for histotype and grading of bladder tumors in cattle [9]. Additionally, four bladders, placenta, fetal liver, kidney, heart, and lung samples were collected from slaughtered healthy pregnant cows. Immunohistochemistry was performed with a rabbit polyclonal anti-E5 antiserum recognizing the C-terminal 14 amino acids of the BPV E5 protein (kind gift of Prof. DiMaio, Yale University School of Medicine, New Haven, CT, USA), as previously described [12]. Antibody specificity was demonstrated by using sections from the same pathological tissue samples where the anti-E5 rabbit polyclonal antiserum was omitted and replaced by a normal rabbit serum (Vector Laboratories Inc., Newark, CA, USA). The owners informed us that the gestational age of these cows was around five months. Both healthy and diseased cows belonged to Podolica breed. All these animals grazed on lands infested with bracken fern (*P. aquilinum*) known as “endemic areas” in mountainous regions of Basilicata and Calabria regions in the southern Italy, prevalently. Bladder tumors were found as high as more than 90% in hematuric cattle from these areas [9]. Only occasionally, chronic enzootic hematuria is found outside these areas. Medical authorities claimed that all these animals were from areas “officially free” from protozoan diseases such as those caused by parasites, collectively called piroplasms, that can be responsible for hematuria in cattle. Finally, no “hematuric” bacteria were identified with bacteriological examinations performed on more than 100 urine samples from cattle affected by papillomavirus infection resulting in chronic enzootic hematuria associated with bladder tumors. 

### 2.3. DNA Extraction

DNA was extracted from blood, bladder, placenta, and fetal organ samples using the DNeasy Blood and Tissue Kit (Qiagen, Wilmington, DE, USA), according to the manufacturer’s instructions.

### 2.4. Droplet Digital Polymerase Chain Reaction (ddPCR)

For ddPCR, (Bio-Rad Laboratories, Hercules, CA, USA) QX100 ddPCR System was used according to the manufacturer’s instructions. The reaction was performed in a final volume of 22 μL, consisting of 11 μL of ddPCR Supermix for Probes (2X; Bio-Rad Laboratories, Hercules, CA, USA), 0.9 μM primer, and 0.25 μM of probe with 7 μL sample DNA corresponding to 100 ng. The tools to generate droplets and a thermal profile have been described previously [25]. The primer and probe sequences for BPV and OaPV were reported in Table 1.

Each sample was analyzed in triplicate to ensure accuracy. Samples with very few positive droplets were reanalyzed to ensure that these low-copy number samples were not due to cross-contamination.

BPV positive controls were BPV1 DNA from a zebra sarcoid (a kind gift from Dr G. Borzacchiello, Dept. Veterinary Medicine and Animal Productions, Naples University), BPV2 DNA clone (a kind gift from Dr A. Venuti, National Cancer Institute “Regina Elena”, Rome), and BPV13 and BPV14 DNAs from bovine bladder tumors from our laboratory. OaPV1- and OaPV2-positive controls were artificially created (IDT, integrated DNA, IA, USA). OaPV3 was a plasmid (vector: pUC19) containing the complete genome. OaPV4 DNA was from a skin cutaneous fibropapilloma of sheep. Both OaPV3 and OaPV4 positive controls were a kind gift from prof. A. Alberti, Dept. Veterinary Medicine, Sassari University).

### 2.5. RNA Extraction and One-Step Reverse Transcription (RT)-ddPCR

RNA was extracted from the above samples using the RNeasy Plus Mini Kit (Qiagen, NW, DE, USA), according to the manufacturer’s instructions. This kit contains genomic DNA (gDNA) eliminator spin columns. One hundred nanograms of RNA was used for the One-Step RT-ddPCR Advanced Kit for Probes (Bio-Rad Laboratories, Hercules, CA, USA), according to the manufacturer’s instructions and as previously described [25]. The One-Step reaction was performed on both samples in which the reverse transcriptase (RT) was added (RT+) and in those without RT (RT-). In the current study, besides DNA, we detected and quantified transcripts of early transforming proteins of the bovine and ovine *Deltapapillomaviruses*, as well as transcripts of L1, the major structural protein of ovine *Dyokappapapillomavirus* in the placenta and fetal organs.

### 2.6. Antibodies

Rabbit polyclonal against constitutive platelet-derived growth factor β receptor (PDGFβR), anti-phosphorylated PDGFβR antibodies, and mouse anti-glyceraldehyde-3-phosphate dehydrogenase (GAPDH) antibody, were purchased from Santa Cruz Biotechnology (Dallas, TX, USA). Rabbit antibody against human embryonic stem cell-expressed Ras (ERAS) (TA324562) was obtained from OriGene Technologies, Inc. (Rockille, MD, USA). Rabbit against both constitutive and phosphorylated AKT antibodies were purchased from Cell Signaling Technology (Leiden, The Netherlands).

### 2.7. Western Blot (WB) and Densitometric Analysis

WB analysis was performed only on samples that tested positive for virus transcripts. Fifty micrograms of extracted proteins were boiled and electrophoresed for 1.5 h at 150 V on a 10% (*w*/*v*) polyacrylamide/SDS gel, as reported previously [25]. The blots were then washed and visualized through enhanced chemiluminescence.

Western blot bands were analyzed and the average intensity of the bands was determined using software (Bio-Rad Laboratories, Hercules, CA, USA). Data were collected in terms of average intensity of bands of ERAS, PDGFβR, and AKT per average intensity of bands of GAPDH and imported to a spreadsheet (Excel; Microsoft, Redmond, WA, USA).

### 2.8. Statistical Analysis

Data pertaining to band intensities for the Western blotting are presented as the mean ± standard deviation (SD). Data were assessed by one-way analysis of variance (ANOVA), followed by Tukey’s test for multiple comparisons of means using the GraphPad PRISM software version 9 (GraphPad Software, San Diego, CA, USA). A *p*-value ≤ 0.05 indicated statistical significance.

## 3. Results

A virological assessment of bladder tumors was performed by detecting and quantifying BPV and OaPV DNA and their transcript levels. A peculiar feature of the bladder samples was the detection of both a BPV and OaPV coinfection. In three bladder samples from two high-grade papillary carcinoma (animals 1 and 4) (Supplemental Figure S1) as well as invasive high-grade carcinoma (animal 3) (Supplemental Figure S2), the viral infection was characterized by the simultaneous presence of all BPV and OaPV genotypes; in only one sample, representing an in situ carcinoma (animal 2) (Supplemental Figure S3), the viral coinfection was composed of four OaPVs and three BPVs as ddPCR failed to detect the presence of BPV1 DNA. Many BPVs and OaPVs were found to be transcriptionally active, as shown by the detection and quantification of their transcript levels. In blood samples from these hematuric pregnant cows, only BPV DNA was found through ddPCR analysis, and no OaPV DNA or any viral transcripts were detected. BPV and OaPV coinfection was also detected in all examined placentas. Supplemental Figure S4 provides results of DNA and messenger RNA (mRNA) copies/µL of BPV and OaPV genotypes in bladder, blood, and placenta samples. A virological examination performed on healthy tissues failed to reveal the presence of any viral DNA and mRNA. Supplemental Figure S5 provides a summary of the detected and quantified BPV and OaPV DNA and its transcripts in fetal organs, such as the liver and kidneys, from three fetuses (samples 1, 2, and 4, respectively) and in the lungs and hearts from two fetuses (samples 1 and 2). Fetuses 1, 2, 3, and 4 are from mothers 1, 2, 3, and 4, respectively. Supplemental Figures S6 and S7 show the immunohistochemical localization of the BPV E5 oncoprotein in placenta and urothelial cancer cells.

**Figure 1:**
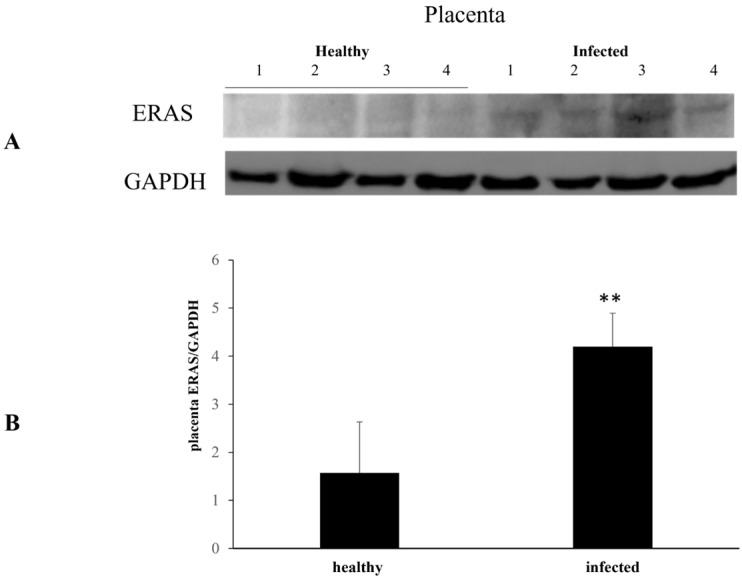
ERAS expression levels in healthy and infected placentas. (**A**) Western blot analysis of ERAS in four healthy and four infected placenta samples and (**B**) densitometric analysis of ERAS relative to GAPDH protein level. Significant ERAS overexpression is evident in infected placentas (** *p* ≤ 0.01). Histograms represent value found in four grouped samples. Each of the samples was analyzed in triplicate.

WB analysis detected a significant increase in ERAS expression levels across all placenta samples (Figure 1).

**Figure 2:**
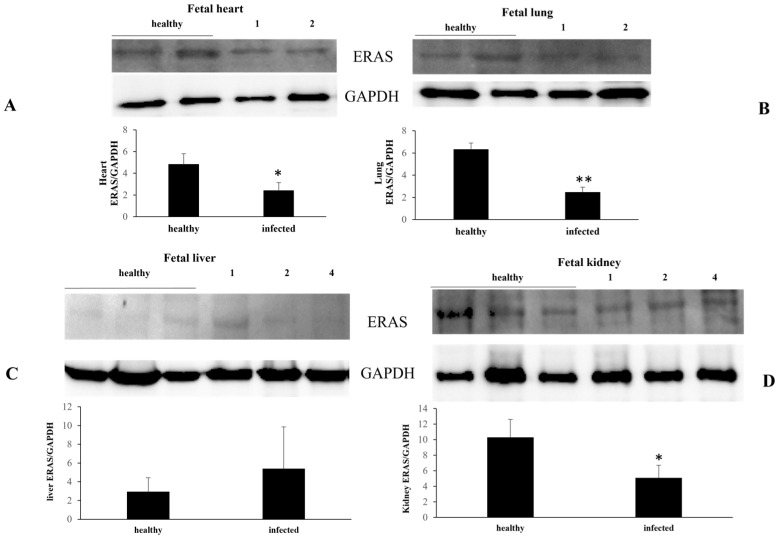
ERAS expression in heathy and infected fetal organs. Western blot and densitometric analysis of ERAS expression levels. Significant ERAS downregulation is shown in infected fetal heart (**A**), lung (**B**), and kidney (**C**) samples in comparison with corresponding healthy fetal organs. In a liver sample there is significant overexpression. No variation is evident in the remaining liver samples (**D**). (* *p* ≤ 0.05 and ** *p* ≤ 0.01). Histograms represent value found in grouped samples. Each sample was analyzed in triplicate.

However, in infected fetal organs, such as the heart, lungs, and kidneys, ERAS expression levels were found to be unstable with a significant decrease. No variation in ERAS expression was detected in two liver samples whereas, in one sample, a significant increase in ERAS levels was observed (Figure 2).

**Figure 3:**
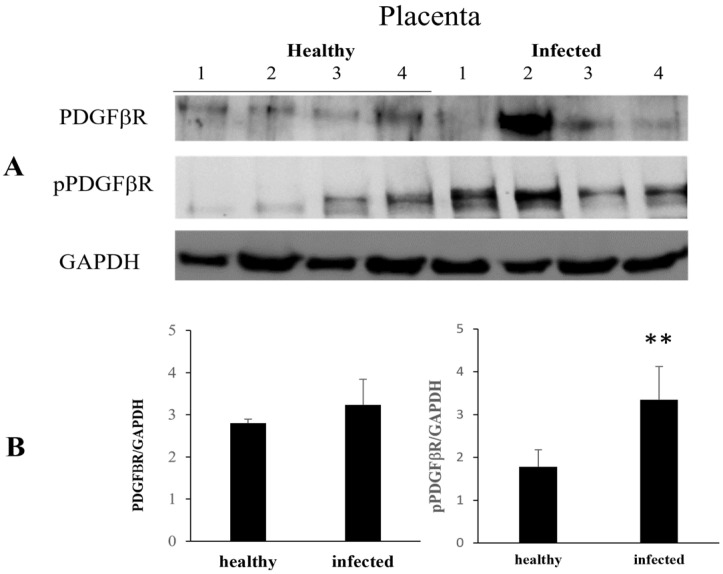
PDGFβR and pPDGFβR expression levels in healthy and infected placentas. (**A**) Western blot and (**B**) densitometric analysis of PDGFβR and pPDGFβR in healthy and infected placenta samples. pPDGFβR protein levels show a significant increase in infected placentas compared to healthy sample (** *p* ≤ 0.01). Histograms represent value found in grouped samples. Each sample was analyzed in triplicate.

WB analysis failed to reveal any significant variation in the constitutive PDGFβR expression at the placenta level. However, immunoblotting showed changes in post-translational modification with a significant increase in PDGFβR phosphorylation (Figure 3).

**Figure 4:**
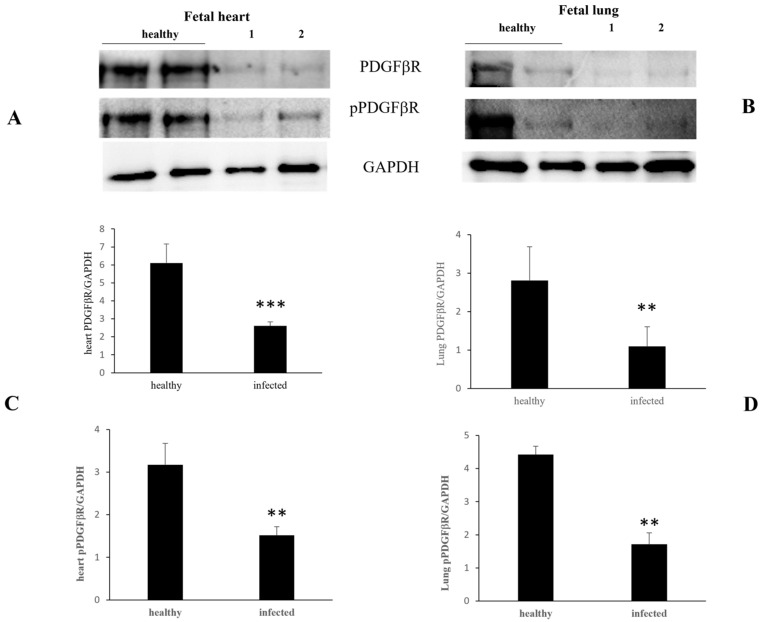
PDGFβR and pPDGFβR expression levels in healthy and infected fetal heart and lungs. Western blot (**A**,**B**) and densitometric analysis (**C**,**D**) show a significant decrease in PDGFβR and pPDGFβR expression in infected fetal heart (**A**,**C**) and lung (**B**,**D**) samples compared to healthy. (** *p* ≤ 0.01 and *** *p* ≤ 0.001). Histograms represent value found in grouped samples. Each sample was analyzed in triplicate.

In the heart and lungs, both constitutive and activated PDGFβR expression levels were significantly decreased (Figure 4).

**Figure 5:**
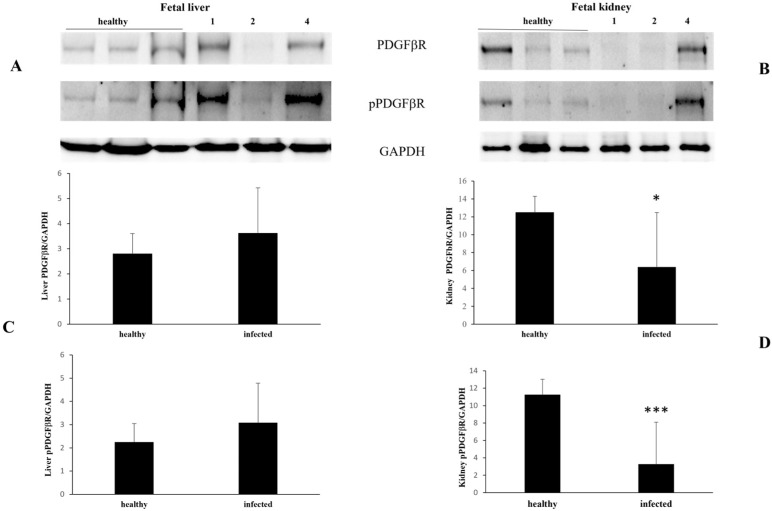
PDGFβR and pPDGFβR expression levels in healthy and infected fetal liver and kidneys. Western blot analysis of PDGFβR and pPDGFβR in healthy and infected fetal liver (**A**) and kidney (**B**) samples. Densitometric analysis of PDGFβR and pPDGFβR proteins (**C**,**D**) shows significant variation in infected fetal organs (* *p* ≤ 0.01, *** *p* ≤ 0.001). Histograms represent value found in grouped samples. Each sample was analyzed in triplicate.

Expression of these proteins was significantly reduced also in two kidney samples, whereas no variation was observed in the remaining kidney sample. A significant over-expression of both the constitutive and phosphorylated PDGFβR components was observed in two liver samples, whereas these components were found significantly reduced in one sample (Figure 5).

**Figure 6:**
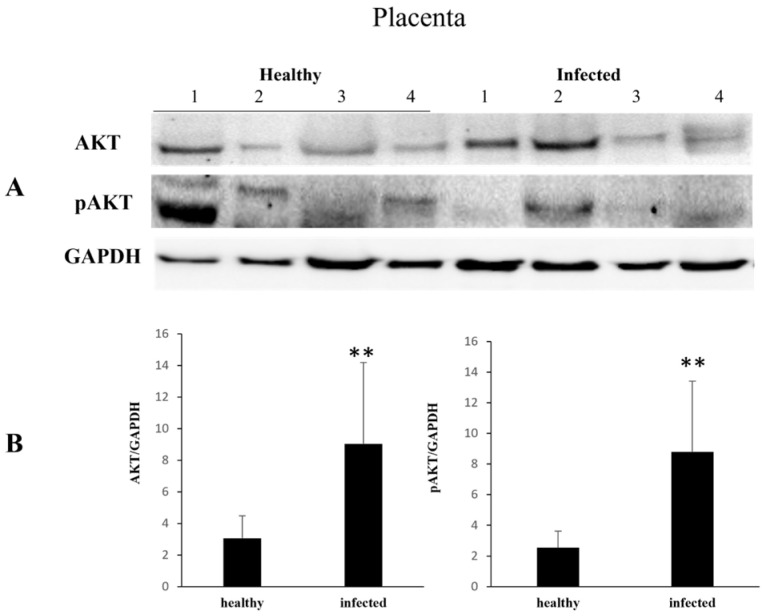
AKT and pAKT expression levels in healthy and infected placentas. (**A**) Western blot analysis of AKT and pAKT in healthy and infected placenta samples and (**B**) their densitometry. Significant increase in expression levels of these proteins is evident in all placental samples (** *p* ≤ 0.01). Histograms represent value found in grouped samples. Each sample was analyzed in triplicate.

Finally, both constitutive and phosphorylated placental AKT proteins were found significantly over-expressed (Figure 6).

**Figure 7:**
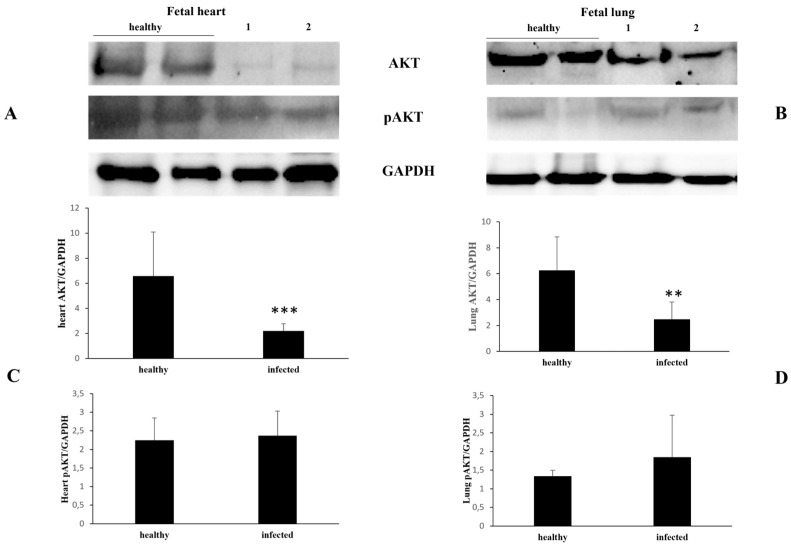
AKT and pAKT expression levels in healthy and infected fetal heart and lungs. (**A**,**B**) Western blot analysis of AKT and pAKT in healthy and infected fetal heart (**A**) and lung (**B**) samples in (**C**) and (**D**)’s densitometric study (** *p* ≤ 0.01 and *** *p* ≤ 0.001). Histograms represent value found in grouped samples. Each sample was analyzed in triplicate.

There was a significant decrease in the constitutive component of the AKT protein observed in the fetal heart and lungs (Figure 7).

**Figure 8:**
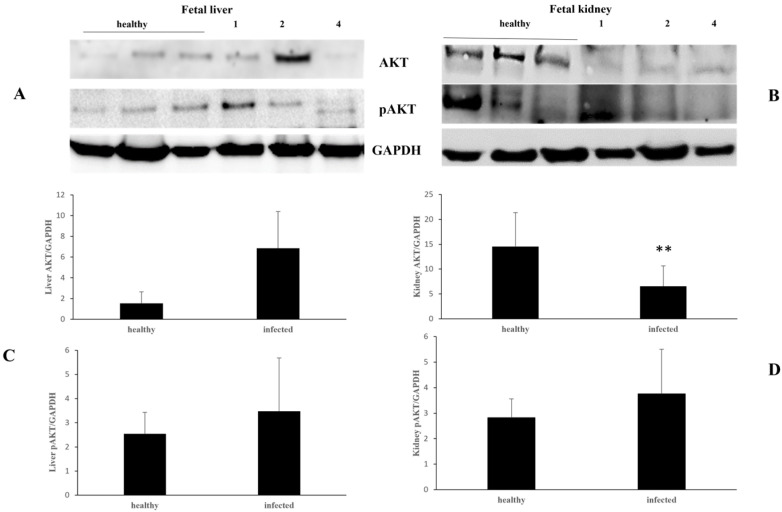
AKT and pAKT expression levels in healthy and infected fetal liver and kidneys. (**A**,**B**). Western blot analysis of AKT and pAKT in healthy and infected fetal liver (**A**) and kidney (**B**) samples with their densitometry, respectively (**C**,**D**). (** *p* ≤ 0.01). Histograms represent value found in grouped samples. Each sample was analyzed in triplicate.

This component was significantly reduced also in kidneys and increased in the liver of two fetuses (Figure 8).

Immunoblotting analysis showed that the activated AKT was significantly increased in the liver and kidneys of two fetuses, as well as in a lung sample (Figure 7 and Figure 8).

## 4. Discussion

This study presents evidence of a novel vertical, congenital coinfection in bovine fetuses by BPVs and OaPVs, which was found in hematuric, pregnant cows affected by BPV- and OaPV-associated tumors of the urinary bladder. The investigation corroborates previous studies on prenatal, vertical infections by BPVs [11] and reports the first congenital cross-species transmission of OaPVs in bovine fetuses. This study, as well as our previous studies, show that OaPVs may have many biological properties similar to those of BPVs being able to cause cross-species transmission and infection [24,25].

The methodological approach of the current study was carried out through the ddPCR tool, which is the most accurate and sensitive method for quantifying nucleic acids of papillomavirus both in large and small ruminants. The diagnostic approach of our study was able to detect a very small amount (<1 copy number/μL) of both viral DNA and mRNA in the placenta, as well as fetal organs. As a highly sensitive procedure, ddPCR is capable of detecting BPV and OaPV nucleic acids that may otherwise be undetectable. Therefore, ddPCR appears to be very useful in the detection and definition of the prevalence and genotype distribution of papillomaviruses in the livestock production system, which could reveal additional roles, if any, of papillomaviruses in infected pregnant cows.

Despite the growing number of pathogens associated with in utero fetal disease, the mechanisms of the vertical transmission of infection across the placental barrier remains largely unknown [32]. While intrauterine infection by bacteria is well established as a pathway leading to spontaneous reproductive disorders, there is still much to learn about the impact of viral infection and pregnancy outcomes [33]. The importance of understanding the role of viral infection during pregnancy is becoming more and more relevant [34]. Viruses rarely cross the placental barrier [35], even if the ability of several viral pathogens to cross the bovine placenta is known [36].

To better understand the risk of the vertical transmission of papillomavirus genotypes and ultimately explain the causality of viral disease, including adverse pregnancy outcomes, it is important to distinguish between markers of inert papillomaviral DNA and markers of active virus infection [33,37]. Besides DNA, we detected BPV and OaPV mRNA, a peculiar marker of active infections, thus showing an actual virus coinfection and refuting any possible contamination, which might be a risk of false positives through very sensitive molecular tools.

Several routes of the vertical transmission of pathogens across the maternal–fetal interface are known to occur, including direct transplacental transmission, placental disruption, fetal–maternal hemorrhage, transmission across fetal membranes, and ascending infections [38,39]. The presence of viral components in the placenta may be due to the translocation of viral components from peripheral blood or viral replication in the placenta [40]. We previously showed that BPVs have the potential to replicate in the placenta as we detected BPV L1 expression in the binucleate trophoblast cells of the placenta from pregnant cows [15]. Therefore, it is conceivable that OaPVs also have the potential to replicate in trophoblast cells, causing both an abortive and productive infection, as BPVs do. It has been shown that BPVs and OaPVs can spread via the bloodstream [24,41,42]. It has been suggested that the blood infected with the papillomavirus yields infections at permissive sites with detectable viral DNA and its transcripts [24,25,43].

Our findings show that there is a stronger concordance between bovine and ovine PV genotypes found in bladders, placenta, and fetal organs than those detected in peripheral blood. Although the mechanisms of the transplacental transfer of BPVs and OaPVs are largely uncharacterized, our findings may suggest that papillomaviruses causing congenital infections may join the placenta by ascending the urogenital tract rather than hematogenous dissemination. It is worth noting that PCR analysis detected virus presence in tissues such as the cervix and vagina. It has been suggested that the genotype PV concordance is crucial to better understand their likelihood as the source and potential modes of viral transmission [44]. The genotype-specific BPV and OaPV concordance was defined when both mother and its fetus tested positive for the specific BPV and OaPV genotypes.

According to Suominen et al. [45], the genotype-specific PV concordance between mother and fetuses is suggestive for vertical intrauterine transmission. It has been suggested that vertical transmission to calves may be linked to the maternal viral load and the higher the viral load is, the higher the risk of its vertical transmission [46]. We detected a low number of copy/μL of BPV and OaPV DNA and mRNA in both the placenta and internal organs of bovine fetuses, thereby suggesting that vertical transmission by BPV and OaPV could be independent in a maternal viral load.

It is believed that placental and fetal growth is also linked to epigenetic mechanisms, including protein phosphorylation, which modulate tissue-specific gene expression [47]. It is conceivable that the abnormal expression of ERAS reflects on some aspects of the innate immunity system of the placenta and the phenotypic pattern of fetal organs. ERAS plays a crucial role in cellular proteostasis as it has been shown to be a key player in chaperone-assisted selective autophagy, as well as Parkin-dependent and -independent mitophagy in cattle and horses [26,30]. It is well known that autophagy plays an important role in the normal development and function of trophoblast cells and is precisely regulated during pregnancy. Dysregulated autophagy may contribute to the occurrence of pregnancy-related diseases [48]. Furthermore, ERAS plays an important role in regulating the state of embryonic stem cells (ESCs) [49], the reprogramming of somatic cells [50], and may be an important target for the differentiation of ESCs into specific lineage cells during embryonic development [51]. Additionally, ERAS plays a key role in the maintenance of quiescent hepatic stellate cells and liver development [52].

Ultimately, regulated PDGFβR signaling is necessary for placental angiogenesis via the recruitment of placental mesenchymal stem cells [28], normal cardiovascular development [53], and kidney and lung development [54]. It is also plausible that abnormal PDGFβR signaling may be responsible for organogenesis defects, including placental, cardiac, renal, and lung abnormalities, as shown by experimental studies [55]. Furthermore, recent in vitro studies showed that PDGFβR signaling regulates mitophagy [56,57]. Therefore, deregulated PDGFβR expression in our study is consistent with the assumption that PDGFβR signaling could be imbalanced, which might reflect on the innate immune response of the placenta and fetuses.

ERAS and PDGFβR share important molecular signaling pathways via the protein kinase AKT, which is a downstream effector. This has been demonstrated in the healthy, full-term placenta of pregnant cows [26], papillomavirus-associated bladder tumors of cattle [30], and in several healthy tissues of adult horses [30]. Activated AKT plays a key role in cellular processes involved in cell survival, growth, and proliferation [58]. Although AKT functions may vary depending on the cell and tissue context [59], phosphorylated AKT may be involved in placental homeostasis, as it is well known that activated AKT regulates trophoblast cell function [60].

## 5. Conclusions

In conclusion, our study highlighted the first report of an intrauterine vertical mixed infection by BPVs and OaPVs in a limited number of cases. This coinfection emphasizes the need to gain insights into the mechanisms and consequences of viral exposure. Further research is required as viral diseases remain a major source of economic losses in the cattle industry [39,61].

## Figures and Tables

**Table 1:** Primers and probes used for the detection of BPVs and OaPVs.

	Forward 5′ 3′	Reverse 5′ 3′	Probe (FAM)	Region
BPV1	ACTTCTGATCACTGCCATT	ATAGAAACCATAGATTTGGCA	TGAAGTGTTTCTGTTTGTGA	ORF E5
BPV2	TACAGGTCTGCCCTTTTAAT	AACAGTAAACAAATCAAATCCA	AACAACAAAGCCAGTAACC	3′UTR E5
BPV13	CTGTGTGGATTTGATTTGTT	CAGGGGGAATACAAATTCT	TGAAGTGTTTCTGTTTGTGA	3′UTR E5
BPV14	CTTTGTTATTGTATATGAGTCTGT	ACTCTTGACGGTTTAAAAGTA	ATCTTGCCAGTGATCCTG	3′UTR E5
OaPV1	CCTGATTCTATGACTGTAAGAGGC	CTCCCCACAGAAGTCCAAG	TGCAACAGCAGAGTCCCATCAGAAG	ORF E5
OaPV2	AGTTCCCGCTCTGATTTACC	ATGGCGGACGTATACTTGTTC	ATTGCCAGCAGTCTCCTCAGTCATTC	L1
OaPV3	AGCCCACACTCCCTGATATAG	TTCAGTCTTTGACAGCACCTC	AGCAACCAGCACTGTACACGCTAT	E7
OaPV4	GGGTTCTATGGTGTCTGCTTAG	GCTCAAAATGGTCTACTGTTGC	CAGGAATGCTCTGTGCAGGGTATAGTG	E5

## Data Availability

All data generated or analyzed during this study are included in this published article.

## Supplementary Materials

The following supporting information can be downloaded at: https://www.mdpi.com/article/10.3390/pathogens13060453/s1, Supplemental Figure S1. **Bladder neoplasia**. Microscopic pattern consistent with papillary carcinoma, high grade. Hematoxylin and eosin. 40X. Supplemental Figure S2. **Bladder neoplasia**. Microscopic pattern consistent with invasive carcinoma, high grade. Hematoxylin and eosin. 40X. Supplemental Figure S3. **Bladder neoplasia**. Carcinoma in situ (CIS), pagetoid variant. Hematoxylin and eosin. 40X. Supplemental Figure S4. provides results of DNA and messenger RNA (mRNA) copies/µL of BPV and OaPV genotypes in bladder, blood, and placenta samples. Virological examination performed on healthy tissues failed to reveal the presence of any viral DNA and mRNA. Supplemental Figure S5. provides a summary of the detected and quantified BPV and OaPV DNA and its transcripts in fetal organs, such as the liver and kidneys. Supplemental Figure S6. **Placenta**. Immunohistochemical localization of BPV E5 oncoprotein in placental cells. Hematoxylin and eosin. 40X. Supplemental Figure S7. **Bladder neoplasia**. Immunohistochemical localization of BPV E5 oncoprotein in urothelial cancer cells. Hematoxylin and eosin. 40X.
