# Supplementary material for: Vertical Intrauterine Bovine and Ovine Papillomavirus Coinfection in Pregnant Cows
Source: Pathogens. 2024 May 26;13(6):453. doi: 10.3390/pathogens13060453 (PMC11206582; doi:10.3390/pathogens13060453)
Supplement: Supplementary file 1 [file pathogens-13-00453-s001.zip › pathogens-2982750-supplementary.pdf]

Supplemental Figure S1. **Bladder neoplasia.** Microscopic pattern consistent with papillary carcinoma, high grade. Hematoxylin and eosin. 40X

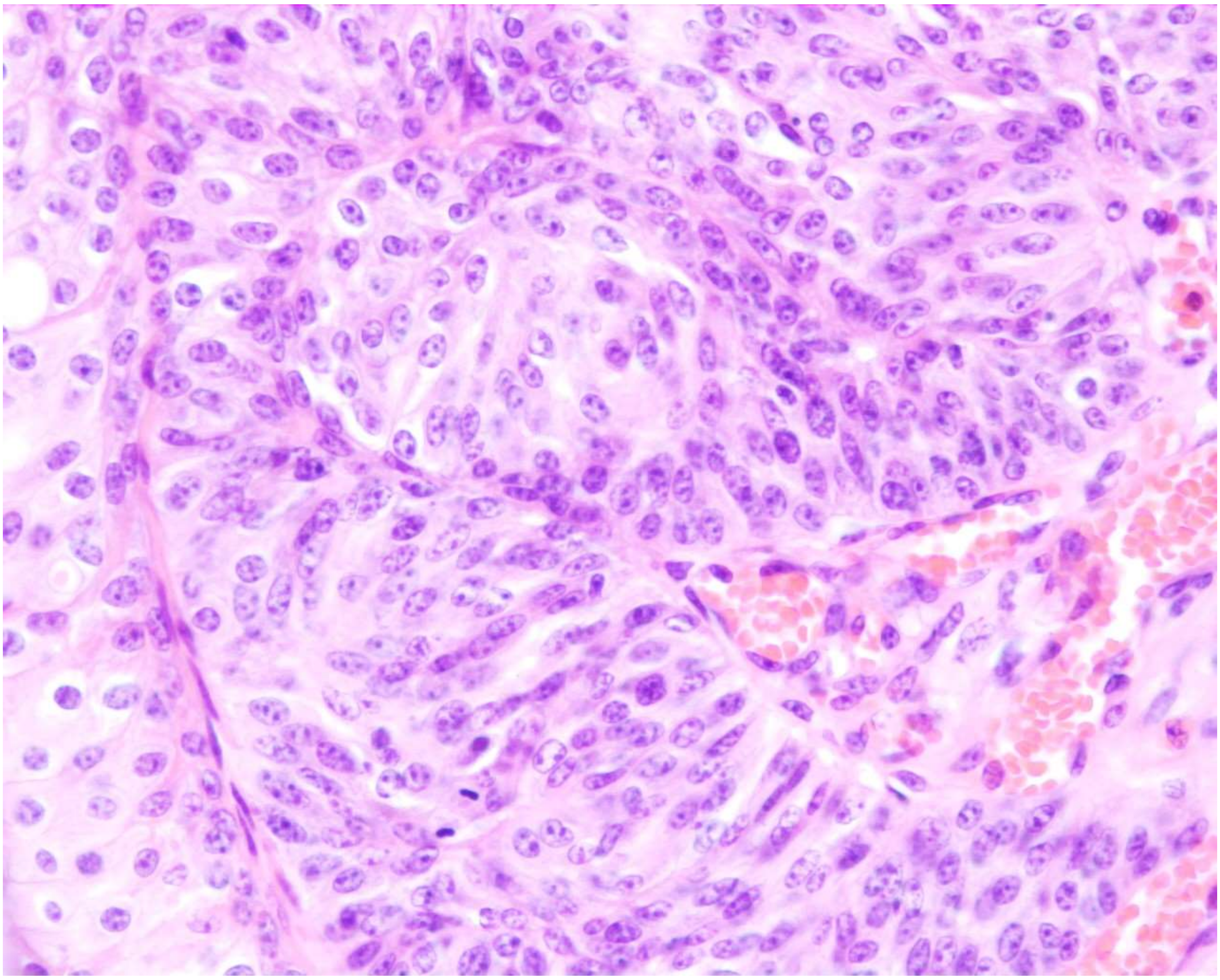

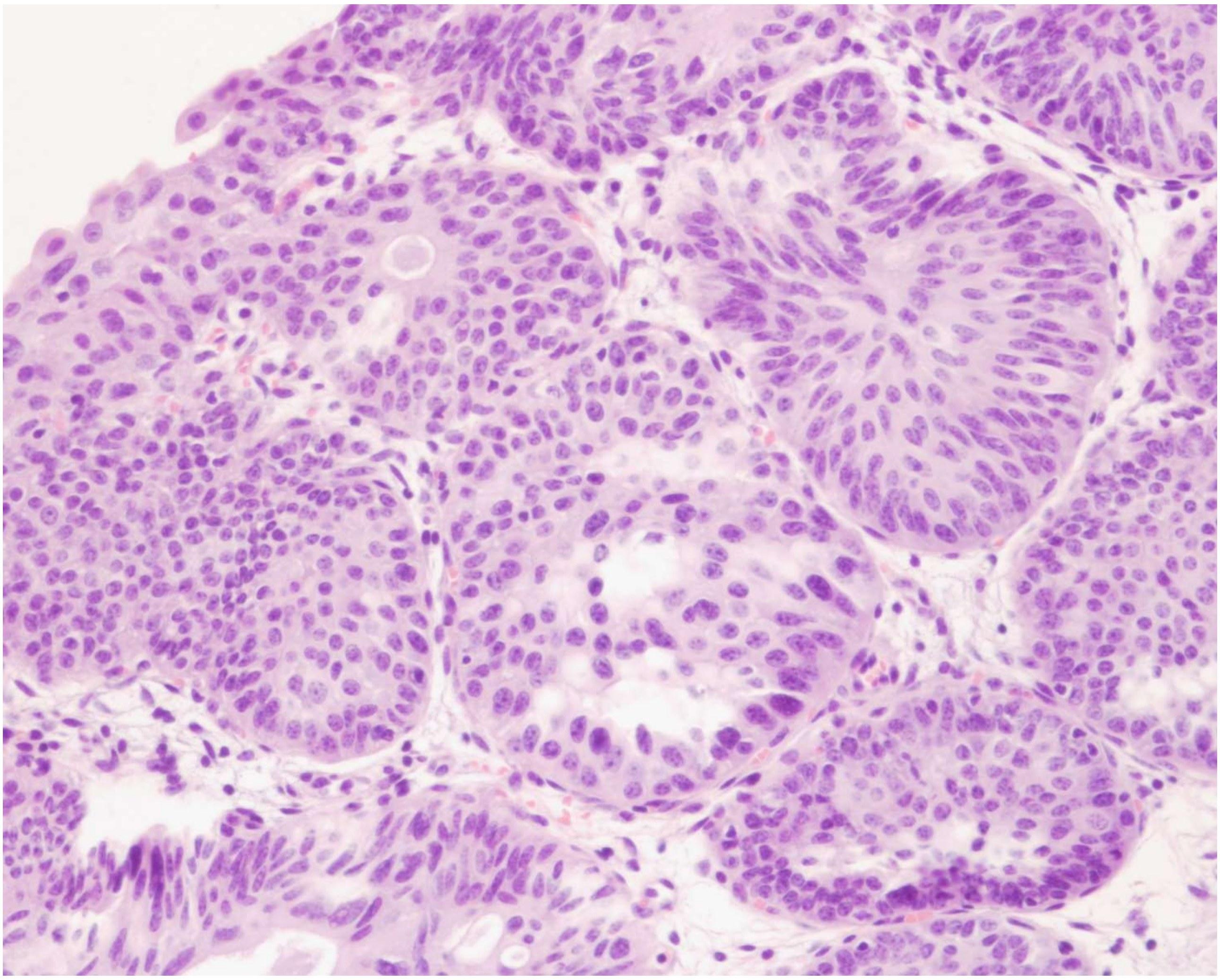

Supplemental Figure S2. **Bladder neoplasia**. Microscopic pattern consistent with invasive carcinoma, high grade. Hematoxylin and eosin. 40X

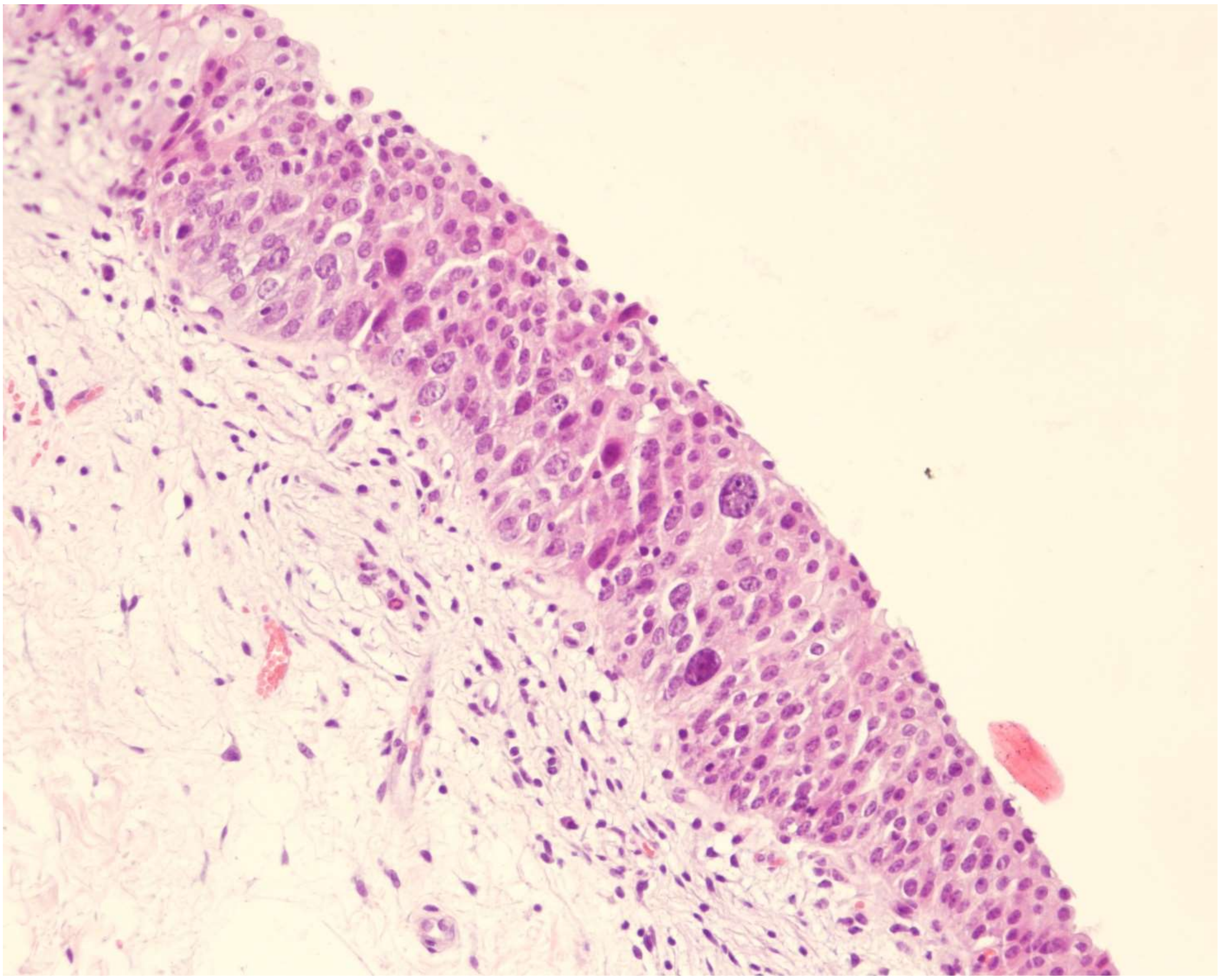

Supplemental Figure S3. Bladder neoplasia. Carcinoma in situ (CIS), pagetoid variant. Hematoxylin and eosin. 40X

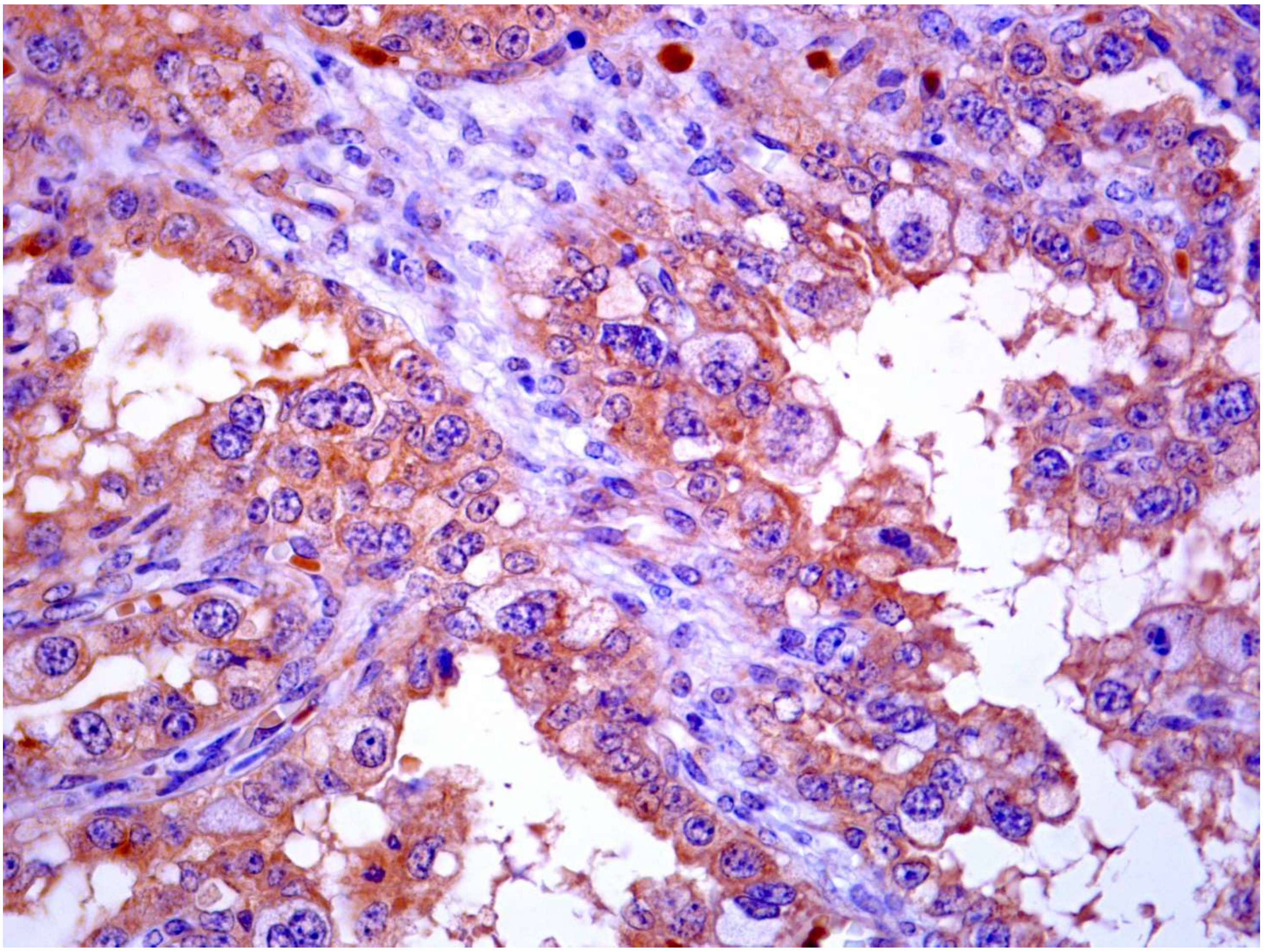

Supplemental Figure S4. provides results of DNA and messenger RNA (mRNA) copies/µL of BPV and OaPV genotypes in bladder, blood, and placenta samples. Virological examination performed on healthy tissues failed to reveal the presence of any viral DNA and mRNA.

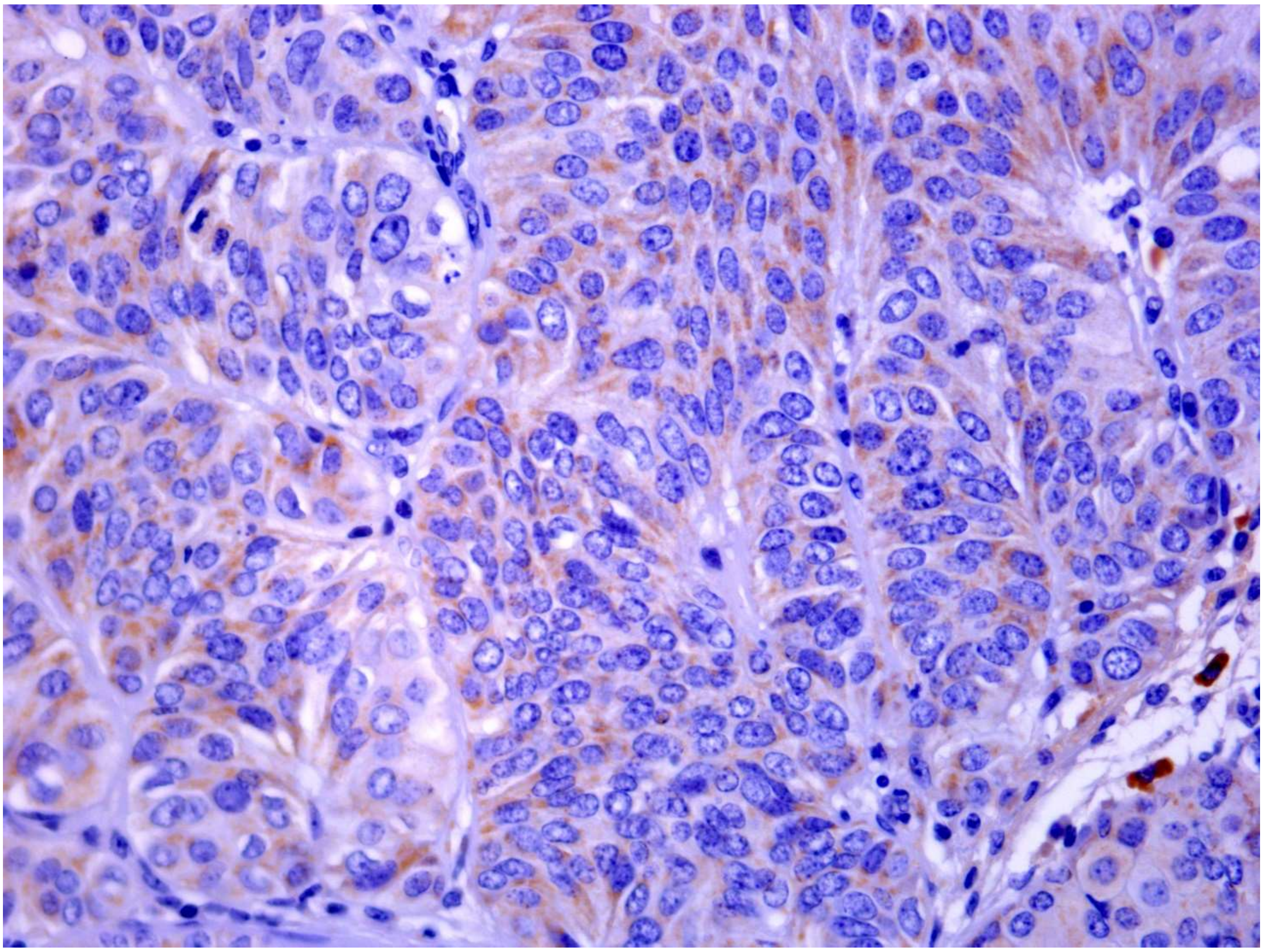

Supplemental Figure S5. provides a summary of the detected and quantified BPV and OaPV DNA and its transcripts in fetal organs, such as the liver and kidneys

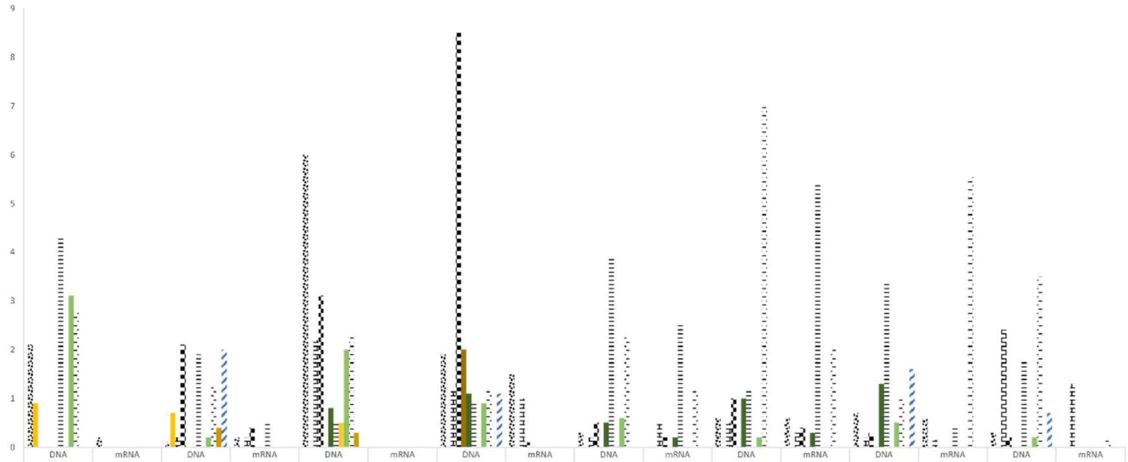

	BPV1		BPV2		BPV13		BPV14		OaPV1		OaPV2		OaPV3		OaPV4	
	DNA	mRNA	DNA	mRNA	DNA	mRNA	DNA	mRNA	DNA	mRNA	DNA	mRNA	DNA	mRNA	DNA	mRNA
1 Bladder	2,1	0,2	0,1	0,2	6		1,9	1,5	0,3		0,6	0,6	0,7	0,6	0,3	
1 Blood	0,9		0,7													
1 Placenta			0,2	0,2	2,2		1,2	1	0,2	0,5	0,5	0,3	0,2	0,15	2,4	1,3
2 Bladder			2,1	0,4	3,1		8,5	0,1	0,5	0,2	1	0,4	0,3		0,2	
2 Blood							2									
2 Placenta					0,8		1,1		0,5	0,2	1	0,3	1,3			
3 Bladder	4,3		1,9	0,5	0,5		0,9		3,9	2,5	1,2	5,4	3,4	0,4	1,8	
3 Blood					0,5											
3 Placenta	3,1		0,2		2		0,9		0,6		0,2		0,5		0,2	
4 Bladder	2,8		1,3		2,3		1,2		2,3	1,2	7	2	1	5,6	3,5	0,13
4 Blood			0,4		0,3											
4 Placenta			2				1,1						1,6		0,7	

Supplemental Figure S6. **Placenta.** Immunohistochemical localization of BPV E5 oncoprotein in placental cells. Hematoxylin and eosin. 40X

Supplemental Figure S7. Bladder neoplasia. Immunohistochemical localization of BPV E5 oncoprotein in urothelial cancer cells. Hematoxylin and eosin. 40X

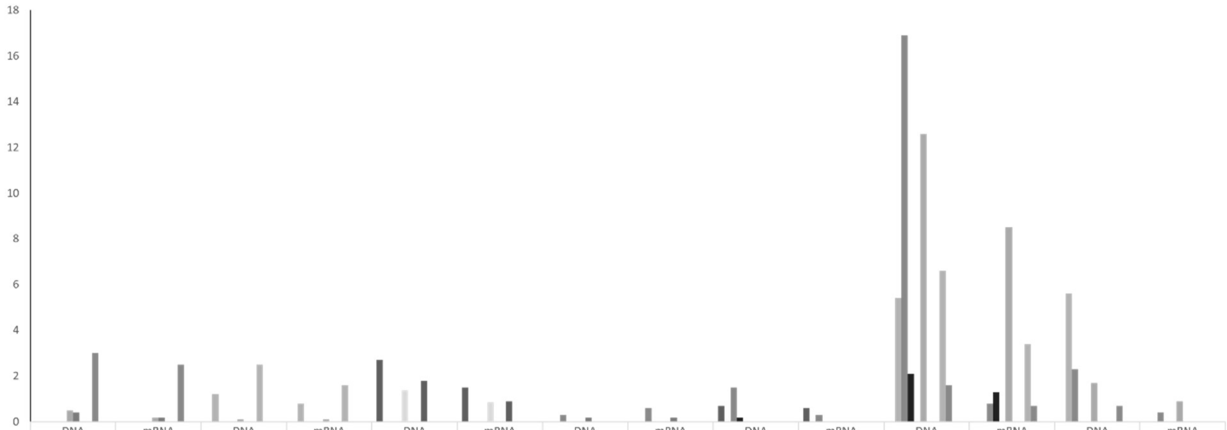

	BPV2		BPV13		BPV14		OaPV1		OaPV2		OaPV3		OaPV4	
	DNA	mRNA	DNA	mRNA	DNA	mRNA	DNA	mRNA	DNA	mRNA	DNA	mRNA	DNA	mRNA
1 Liver					2,7	1,5			0,7	0,6				
1 Kidney			1,2	0,8							5,4		5,6	
1 Heart							0,3	0,6	1,5	0,3	16,9	0,8	2,3	0,4
1 Lung									0,2		2,1	1,3		
2 Liver					1,39	0,85								
2 Kidney	0,5	0,2	0,1	0,1							12,6	8,5	1,7	0,9
2 Heart	0,4	0,2					0,2	0,2						
2 Lung					1,8	0,9								
4 Liver			2,5	1,6							6,6	3,4		
4 Kidney	3	2,5									1,6	0,7	0,7	
4 Heart														
4 Lung														
